# Nurses’ needs for digital mental health support: an analysis based on the Kano model

**DOI:** 10.3389/fpubh.2026.1796306

**Published:** 2026-04-21

**Authors:** Zhongqing Chen, Peng Xu, Zhanghao Xie, Ziyi Xiong, Xuanhao Fan, Jiehao Zhuang, Niu Yang, Tiemei Shen, Lifang Chen, Huigen Huang

**Affiliations:** 1 Department of Nursing, Guangdong Provincial People’s Hospital, Guangdong Academy of Medical Sciences, Southern Medical University, Guangzhou, China; 2 School of Nursing, Southern Medical University, Guangzhou, China; 3 Department of Nursing, Zhongshan Ophthalmic Center, Sun Yat-sen University, Guangzhou, China; 4 School of Nursing, Guangdong Pharmaceutical University, Guangzhou, China; 5 School of Nursing, Jinan University, Guangzhou, China

**Keywords:** demand analysis, digital support, Kano model, mental health, nurses

## Abstract

**Objective:**

Nurses work under high pressure for a long time and are prone to anxiety, depression and other adverse emotions. Due to the nature of their work as well as time and space constraints, it is often difficult for them to pay attention to their own mental health problems in a timely manner. Digital mental health support can give nurses support to a certain extent and effectively relieve their stress. The aim of this study is to investigate and analyze nurses’ demand for digital mental health support, to provide reference for the development of digital psychological interventions, and to provide a decision-making basis for the construction of a precise psychological support system and digital platform.

**Methods:**

This study employed the Kano model as an analytical tool to classify nurses’ needs for digital mental health support. A cross-sectional survey was conducted with 1,571 nurses using a questionnaire developed through literature review and semi-structured interviews. The questionnaire included 26 items representing potential digital mental health features. For each item, participants answered paired functional and dysfunctional questions. Responses were classified into must-be, one-dimensional, attractive, and indifferent attributes using the standard Kano evaluation table. These demand attributes were then combined with the importance-satisfaction matrix to quantify the sensitivity of demand improvement.

**Results:**

Of the 26 needs, 8 were classified as must-be attributes, 8 as one-dimensional attributes, 7 as attractive attributes and 3 as indifferent attributes. Nurses rated the importance index of the digital mental health support demand items from −0.62 to −0.15, and satisfaction index from 0.34 to 0.62.

**Conclusion:**

The KANO model was applied to identify and categorize the different attributes of nurses’ digital psychological support needs, offering targeted recommendations and strategies to address these needs, thereby providing a foundation for digital psychological interventions for nurses. Nursing managers can combine the results of Kano model analysis in the importance-satisfaction matrix to improve and satisfy nurses’ demand for psychological support, so as to reduce their psychological pressure, improve job satisfaction, and lay the foundation for cultivating a high-level nursing team and promoting the high-quality development of nurses’ career.

## 1 Introduction

Against the backdrop of a modern healthcare system operating at high speed, the nursing community is undergoing unprecedented occupational stress. High-intensity work rhythms, such as night shifts (1), complex and changing emergencies, potential risks of occupational violence, and difficulties in balancing work-life relationships, together constitute multiple sources of occupational stress for nurses. This stress has a serious impact on their mental health (2, 3), not only leading to mental health problems such as anxiety and depression among the nursing population, but also having a knock-on effect on the quality of care, patient safety, and the efficiency of nursing management. Especially during the coronavirus disease 2019 (COVID-19) pandemic, the increased workload, risk of infection and other related stressors further exacerbated the burnout, depression and anxiety problems of the nurse population (4–6) making the urgency of improving their mental health more and more prominent.

In recent years, the boom in information technology has opened up new pathways for mental health interventions for nurses (7). Digital interventions refer to interventions that provide information, support, and treatment for physical and/or mental health problems through digital technologies or digital platforms (e.g., websites, computers, mobile apps, text messaging, emails, videoconferencing, wearable devices, etc.) (8). With its significant advantages of convenience, accessibility, and personalization, digital interventions are gradually becoming the focus of academic attention. Digital tools such as mobile-based mental health applications (APPs) and online training platforms can help nurses effectively cope with work stress by providing real-time psychological guidance, intelligent time management systems, and customized professional development courses (9). Several empirical studies have shown that the mental health status of nurses adopting digital intervention programs has been significantly improved (10, 11), which fully validates the practical value of digital intervention in this field.

However, despite the great potential of digital interventions, current research on the needs of digital interventions for nurses’ mental health is still insufficient. Mental health itself encompasses multiple dimensions, which, combined with individual differences in nurses and differences in their organizational environments (10), results in significant heterogeneity in their needs. In this case, the priority of demand elements has not yet been scientifically defined, and the importance of each element in the actual needs of nurses is unknown, which directly leads to the lack of precise targeting of intervention programs, making it difficult to effectively meet the real needs of nurses. This deficiency makes the digital intervention products lack of basis in functional design, resulting in low utilization rate and poor user stickiness in practical application (12), which seriously restricts the effect and sustainable development of digital intervention.

The Kano model, proposed by Professor Noriaki Kano and his colleagues in 1984, is a classic analytical tool for classifying user requirements (13), and provides new ideas to address these challenges. By employing paired functional and dysfunctional questions, the model categorizes needs into six types, must-be attributes, one-dimensional attributes, attractive attributes, indifferent attributes, reverse attributes, questionable attributes (14), according to the relationship between the objective performance of the product and the subjective feelings of the user, and then provides the basis for the formulation of the quality management strategy and the improvement program. The Kano model originated in the field of product development and customer satisfaction (13). In recent years, its application scope has continuously expanded, and it has now been widely applied in digital business contexts to assess user satisfaction and categorize customer requirements for e-commerce platforms, mobile applications, and online services (15–17). Through categorizing and prioritizing needs, the Kano model enables researchers to quantify the service requirements of the study population and identify which attributes warrant focused attention and prioritized improvement, thereby guiding efforts to enhance service quality (18). Since the Kano model was introduced into the nursing field, it has achieved good results in improving the quality of nursing services and enhancing patient satisfaction (19, 20). However, there are limitations of single research object in the application: most of the existing researches focus on the care recipients, and the research on the needs of nurses’ group is obviously insufficient (21). The application of the Kano model to understand nurses’ own service needs is still in its initial stage.

Based on this, the study applies the Kano model to investigate nurses’ needs for digital mental health interventions. Through systematic attribute classification and analysis, this study aims to determine the priority of each need dimension and propose targeted optimization strategies. The findings are expected to provide evidence-based guidance for the development of digital mental health platforms for nurses, thereby contributing to the improvement of their psychological wellbeing. Furthermore, this study may offer new insights for innovation in nursing management models and enhancement of nursing quality, while also addressing the current gap in Kano model applications targeting the nurse population.

## 2 Methods

### 2.1 Study design

This study employed a cross-sectional survey design using the Kano model to classify nurses’ needs for digital mental health support.

### 2.2 Participants

A convenience sample of nurses was recruited from tertiary hospitals in Guangdong Province, China, between March and April 2024. The inclusion criteria were as follows: (1) nurses who have obtained nursing practice qualifications and are officially registered; (2) nurses who have been working in clinical practice for more than 6 months; (3) nurses without mental or cognitive disorders; (4) nurses who voluntarily participate in this study. The exclusion criteria were as follows: nurses who are not on duty due to vacation, further training, etc. This study has been approved by the Ethics Committee of Guangdong Provincial People’s Hospital (ethical approval number: KY2023-1104-02).

According to Kendall’s sampling estimation method, the sample size of the study should be 5–10 times the number of independent variables (22). There were 26 independent variables in this study. Assuming that the non-response rate does not exceed 20%, the required sample size for this study is (26 × 5) × 1.2- (26 × 10) × 1.2; that is, the sample size ranges from 156 to 312 cases.

### 2.3 Questionnaire development

#### 2.3.1 Item generation

##### 2.3.1.1 Literature review

A literature review was first conducted to identify established dimensions of digital mental health support needs among nurses. Databases including PubMed, Web of Science, CINAHL, Scopus, and CNKI were searched for studies published between January 2000 and December 2023. The search terms included combinations of (“digital mental health” OR “mHealth” OR “mobile health”) AND (“nurses” OR “nursing staff” OR “healthcare workers”) AND (“needs assessment” OR “user requirements” OR “user needs” OR “KANO model”). However, the available literature specifically addressing nurses’ needs for mobile mental health applications was limited and offered little guidance on item content. Consequently, the review served primarily as a methodological reference, and the subsequent semi-structured interviews became the main source for item generation.

##### 2.3.1.2 Semi-structured interviews

Given the limited literature, semi-structured interviews were conducted as the primary method for item generation. A total of 15 nurses were recruited from tertiary hospitals in Guangdong Province. The interview guide explored the following areas: (1) participants’ current use of digital mental health resources (e.g., types of tools used, frequency, purposes); (2) perceived functional requirements for a psychological resilience management mobile application (e.g., desired features, content preferences, interface expectations); (3) challenges faced in coping with occupational stress and gaps in existing digital support; and (4) suggestions for improving digital mental health tools to better meet nurses’ needs.

Interviews were audio-recorded with participants’ consent and transcribed verbatim within 24 h. For non-audio-recorded portions, detailed notes were completed immediately based on the researcher’s memory, with nonverbal information integrated into the record. Transcripts were returned to participants for verification to ensure accuracy. Data collection and analysis were conducted simultaneously. Two researchers independently read all transcripts and performed thematic analysis using NVivo 15 software. The analysis proceeded as follows: each transcript was divided into meaningful units and coded according to the research questions to generate initial codes. Codes were then grouped into sub-themes, which were further aggregated into overarching themes. Inter-coder reliability was assessed using Cohen’s kappa coefficient, with a target value of ≥0.80 indicating substantial agreement (23). Disagreements were resolved through discussion between the two researchers until consensus was reached. A third researcher reviewed the final coding framework to ensure consistency and neutrality. This process yielded three core thematic dimensions: mental health service needs, resilience training needs, and basic functional needs, as summarized in Table 1.

**Table 1:** Thematic dimensions and examples of items generated from interviews.

Dimension	Description	Example items
Mental health service needs	Access to professional psychological resources and support	Psychological test result analysis, professional knowledge recommendations, access to online/offline counseling
Resilience training needs	Tools and techniques for building psychological resilience	Mindfulness exercises, emotion regulation training, stress management tools, gratitude practice, mood diary
Basic functional needs	Core platform features that ensure usability and trust	Personal data recording, privacy protection, interface personalization, ease of navigation

#### 2.3.2 Pilot study

A pilot study was conducted with 52 nurses who met the inclusion criteria but were excluded from the main survey. The primary purpose of the pilot study was to assess the clarity, comprehensibility, and usability of the preliminary questionnaire. Participants completed the preliminary questionnaire and provided feedback on item wording, layout, and completion time. Based on pilot feedback, 8 items were reworded for clarity. It should be noted that the pilot study did not test the relevance, reliability, or construct validity of the proposed variables.

#### 2.3.3 Final questionnaire structure

The final questionnaire survey in this study was divided into two parts. The first part was a self-designed general information survey form, which included self-reported gender, age, educational background, marital status, number of children, work location, years of work experience, department, professional title, employment status, and monthly income.

The second part was a questionnaire on nurses’ needs for digital mental health support, consisting of 26 items (see Supplementary material for details). For each item, participants responded to paired functional (“If this feature was available, how would you feel?”) and dysfunctional (“If this feature was NOT available, how would you feel?”) questions using a 5-point Likert scale: 1 = I like it that way, 2 = It must be that way, 3 = I am neutral, 4 = I can live with it that way, 5 = I dislike it that way. The combination of responses to each pair was classified into one of six Kano categories (must-be, one-dimensional, attractive, indifferent, reverse and questionable) using the standard Kano evaluation table (Table 2). The Cronbach’s alpha coefficient for the final questionnaire is 0.905, with a Cronbach’s alpha coefficient of 0.934 for the positive section and a Cronbach’s alpha coefficient of 0.901 for the negative section. It should be noted that the questionnaire was not a pre-validated instrument; the absence of expert content validation is acknowledged as a limitation of this study.

**Table 2:** Kano evaluation table.

Functional form	Dysfunctional form				
	I like it that way	It must be that way	I am neutral	I can live with it that way	I dislike it that way
I like it that way	Q	A	A	A	O
It must be that way	R	I	I	I	M
I am neutral	R	I	I	I	M
I can live with it that way	R	I	I	I	M
I dislike it that way	R	R	R	R	Q

### 2.4 Data collection

The project team’s supervising instructor contacted the heads of nursing departments at various hospitals. After obtaining their consent and cooperation, the questionnaire was distributed to nursing staff at each institution. Three nursing graduate students who had undergone training and assessment were responsible for distributing and collecting the questionnaires. The survey questionnaire was distributed via the Questionnaire Star platform, which provided electronic questionnaire link or two-dimensional code. The platform set the logical connections between questions and limits the number of attempts to answer them, with a time limit of 20 min. A total of 1,723 questionnaires were received. All returned questionnaires were organized, with 68 excluded due to obvious logical errors and 83 excluded due to completion times of less than 3 min. Ultimately, 1,571 valid questionnaires were collected, resulting in a response rate of 91.18%.

### 2.5 Statistical analysis

#### 2.5.1 Descriptive statistics

Data were analyzed with IBM SPSS Statistics 27.0 software. The sociodemographic characteristics of the study subjects were described using frequency and percentage.

#### 2.5.2 Kano classification

Each item was classified into one of six Kano categories using the standard Kano evaluation table (Table 2). The Kano model questionnaire was statistically analyzed using frequency counts, and the most frequently occurring statistical results were used to identify the final demand types.

#### 2.5.3 Importance-satisfaction matrix

Based on the analysis results of Kano model, Importance Index (DSI) and Satisfaction Index (SI) can be calculated, and then an Importance-Satisfaction Matrix diagram can be drawn. The formulas for calculating SI and DSI are as follows: SI = (A + O) / (A + O + M + I); DSI = (−1) × [(O + M) / (A + O + M + I)] (24). When the Importance Index is closer to −1, it means that the item has the greatest impact on the importance of the participant; similarly, the closer the Satisfaction Index is to 1, it means that the item has the greatest impact on the satisfaction of the participant.

Importance-Satisfaction Matrix: The SI is plotted on the vertical axis, and the DSI on the horizontal axis, dividing the matrix into four quadrants, each requiring different response strategies (25). First Quadrant: Predominance Area I. When the needs in this area are not met, patient satisfaction will significantly decline. Second Quadrant: Improving Area II. Patient satisfaction is relatively low in this quadrant, and the indicators within this quadrant are key areas for improvement. Third Quadrant: Secondary Improving Area III. Indicators in this quadrant are not key evaluation metrics, are not important to patients, and have low satisfaction ratings, serving only as observation items. Fourth Quadrant: Reserving Area IV. These factors are not important to patients but have high satisfaction ratings. These factors are crucial for maintaining the status quo.

## 3 Results

### 3.1 General information on the study population

A total of 1,571 nurses from tertiary hospitals in Guangdong Province, China, completed the questionnaire survey. Among them, 68 were male, accounting for 4.33% of the total; 1,503 were female, accounting for 95.67% of the total number of nurses. In terms of educational background, 463 participants (29.47%) had a junior college or below, 1,084 (69.00%) had an undergraduate degree, and 24 (1.53%) had a master’s degree or above; the majority (58.82%) had a junior professional title. The distribution of participants’ sociodemographic characteristics is shown in Table 3.

**Table 3:** Demographic characteristics of participants (*N* = 1,571).

Characteristics	Categories	Observation (*N*)	Percentage (%)
Gender	Male	68	4.33
	Female	1,503	95.67
Age (years)	≤30	669	42.58
	31–40	560	35.65
	>40	342	21.77
Education level	Junior college or below	463	29.47
	Undergraduate	1,084	69.00
	Master’s degree or above	24	1.53
Marital status	Unmarried	481	30.62
	Married	1,050	66.84
	Divorce or Widowhood	40	2.55
Number of children	0	579	36.86
	1	451	28.71
	≥2	541	34.44
Years of service (years)	≤10	776	49.40
	11–20	490	31.19
	>20	305	19.41
Department	Internal medicine	375	23.87
	Surgery	333	21.20
	Obstetrics or gynecology	128	8.15
	Outpatient department	93	5.92
	Pediatrics	84	5.35
	Emergency Department	99	6.30
	Intensive care unit	119	7.57
	others	340	21.64
Professional title	Junior	924	58.82
	Intermediate	507	32.27
	Senior	140	8.91
Employment status	Permanent	517	32.91
	Temporary	1,054	67.09
Monthly income (yuan)	≤5,000	368	23.42
	5,001–10,000	846	53.85
	10,001–15,000	263	16.74
	>1,500	94	5.98

### 3.2 Analysis of nurse demand attributes

After analysis, among the 26 items of digital mental health support needs of nurses surveyed, there were 8 must-be attributes, 8 one-dimensional attributes, 7 attractive attributes, and 3 indifferent attributes. No reverse attributes or questionable attributes were found. The must-be attributes included features related to psychological information analysis and personal data management. The one-dimensional attributes primarily consisted of professional support services and interactive features. The attractive attributes were mainly experiential and mindfulness-based features, while the indifferent attributes included AI interaction and reward mechanisms. The specific classification results are shown in Table 4.

**Table 4:** Kano attributes of nurses’ demand for digital mental health support.

No	Items	Frequency						Attribute	DSI	SI
		M	O	A	I	R	Q			
1	Intelligent emotion monitoring	282	235	725	322	2	5	A	−0.33	0.61
2	Professional psychology courses	235	672	246	413	3	2	O	−0.58	0.59
3	Professional psychology online counseling	258	626	229	453	4	1	O	−0.56	0.55
4	Professional psychology testing	151	582	307	527	3	1	O	−0.47	0.57
5	Psychological test result analysis	654	209	471	220	10	7	M	−0.56	0.44
6	Recommended psychological knowledge	599	279	246	434	8	5	M	−0.56	0.34
7	Psychology articles/educational videos	699	272	291	301	5	3	M	−0.62	0.36
8	Offline psychological counseling	680	122	493	269	5	2	M	−0.51	0.39
9	mindfulness practice	224	130	667	541	7	2	A	−0.23	0.51
10	Emotional regulation training	661	285	375	245	4	1	M	−0.60	0.42
11	Stress relief games	202	628	272	458	7	4	O	−0.53	0.58
12	Gratitude practice	131	223	640	573	1	3	A	−0.23	0.55
13	Muscle relaxation training	188	267	597	512	6	1	A	−0.29	0.55
14	mental journaling	131	102	714	608	10	6	A	−0.15	0.52
15	Light music	240	563	217	537	5	9	O	−0.52	0.50
16	White noise	148	534	433	454	1	1	O	−0.43	0.62
17	Psychological Radio Station	159	185	418	804	2	3	I	−0.22	0.39
18	Personal data recording	680	272	344	266	7	2	M	−0.61	0.39
19	User privacy and security protection	319	640	251	351	6	4	O	−0.61	0.57
20	Nurse confession	223	494	460	388	4	2	O	−0.46	0.61
21	Voice sharing	608	213	356	387	6	1	M	−0.52	0.36
22	AI interaction	281	150	488	643	6	3	I	−0.28	0.41
23	Check-in reward system	298	215	397	652	5	4	I	−0.33	0.39
24	health monitoring	205	243	663	454	4	2	A	−0.29	0.58
25	Program customization	558	184	494	330	4	1	M	−0.47	0.43
26	Live-streaming training	162	126	645	631	3	4	A	−0.18	0.49

### 3.3 Nurses’ demand importance-satisfaction matrix

**Figure 1:**
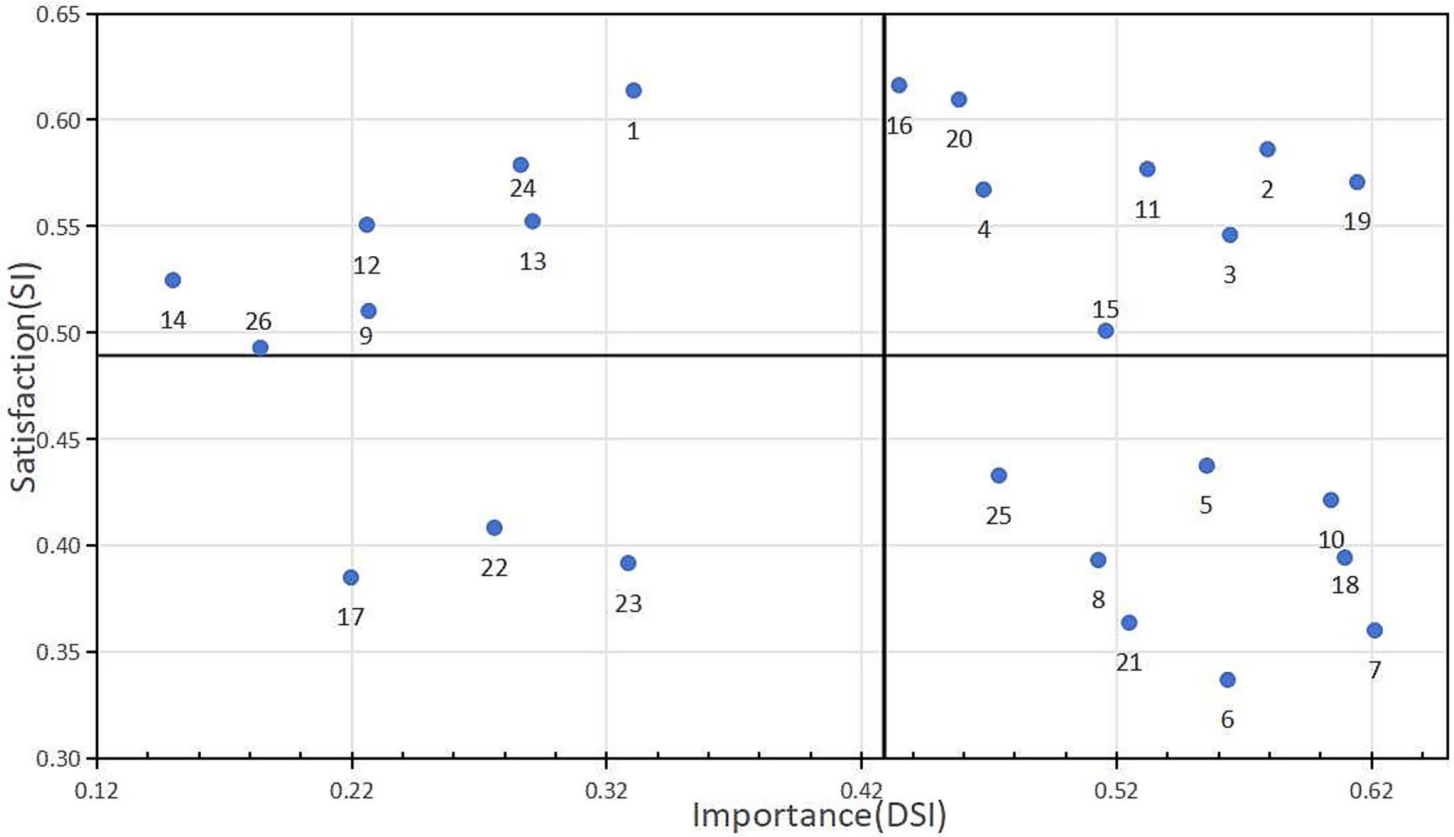
Matrix graph of nurses’ demand importance–satisfaction.

Nurses rated the DSI of the digital mental health support information needs items from −0.62 to −0.15, and SI from 0.34 to 0.62. Based on the calculation results of the DSI and SI values of each entry, the mean value of the Satisfaction Index of the 26 entries was found to be 0.43 and the absolute value of the mean value of the Importance Index was found to be 0.49, respectively, which was used as the origin of the right-angled coordinate system, and then the Importance-satisfaction Matrix was plotted. The first quadrant represents the predominance area I, including 8 items (2, 3, 4, 11, 15, 16, 19, 20). The second quadrant represents improving area II, including 6 items (1, 9, 12 13, 14, 24, 26), the third quadrant is the secondary improving area III, including 3 items (17, 22, 23), and the fourth quadrant is the reserving area IV, including 8 items (5, 6, 7, 8, 10, 18, 21, 25), as shown in Figure 1.

## 4 Discussion

### 4.1 Must-be attributes

Must-be demands are the basic characteristics of services and refer to services that participants take for granted or must have. When this type of need is met, participants’ satisfaction does not increase, but when it is missing, their satisfaction decreases significantly (26). In this study, 8 items were classified as must-be attributes. Based on their nature, these 8 items can be further distinguished into two conceptual categories: digitally-delivered psychological services (6 items) and core platform features (2 items).

Digitally delivered psychological services include psychological test result analysis, psychological knowledge recommendations, psychological articles or educational videos, offline psychological counseling, emotional regulation training, and voice sharing. These needs could theoretically be met through non-digital channels such as face-to-face counseling or printed materials, but are here facilitated by technology. Due to the high-intensity, high-risk and high-stress nature of nursing, nurses often find it difficult to pay attention to and intervene in their own mental health problems in a timely manner (27), so they want to intervene in their own psychological problems with the help of remote and digitally intelligent means. Previous studies have confirmed this view. A survey of nurses’ own health education needs found that most nurses had a high demand for psychological knowledge acquisition (28). It was also found that 64.7% of nurses felt they had a moderate or high demand for psychological counseling (29). Among them, psychological test results analysis, recommended psychological knowledge, psychological articles or educational videos provide nurses with an effective way to acquire psychological-related knowledge; offline psychological counseling appointments and emotional regulation training help nurses regulate negative emotions and reduce psychological pressure. At the same time, studies have shown that voice communication can effectively alleviate loneliness and emotional burden (30). Through voice sharing and communication, Nurses can express their true inner feelings without face-to-face pressure, promote emotional release and obtain psychological support.

Core platform features include personal data recording and program customization. Unlike the psychologically oriented services described above, these items represent essential technical features of the digital platform itself. These attributes have no offline equivalent and are intrinsically tied to the functionality and trustworthiness of the digital system. Personal data recording enables nurses to track their psychological status over time, creating a foundation for self-monitoring and longitudinal awareness of mental health changes. Program customization helps to meet the individual demands of nurses. These features, while not directly psychological in nature, are foundational requirements for any effective digital mental health tool (31), as they determine whether nurses can trust, navigate, and consistently use the platform.

These demands, as must-be demands, were located in the reserving area IV of the Kano model matrix, indicating that while nurses do not perceive these features as directly increasing satisfaction because they are taken for granted, their absence would lead to significant dissatisfaction. Accordingly, it is recommended that nursing administrators provide them in a timely manner and continue to improve them, thus effectively increasing nurses’ motivation to use digital mental health services. These features function as foundational support mechanisms. By providing reliable access to psychological information and personal health data, they reduce uncertainty and establish a baseline of self-awareness (32), enabling nurses to better understand and monitor their own mental state. This foundational support helps nurses recognize early signs of stress and take proactive steps toward self-care (33).

### 4.2 One-dimensional attributes

One-dimensional demands are needs that participants expect to achieve, and the degree to which they are met is directly proportional to participants’ satisfaction (34). Specifically, when these demands are effectively responded to, nurses’ satisfaction increases linearly with the degree of demand fulfillment; conversely, if the demand is not adequately fulfilled, this may raise the risk of decreasing satisfaction. In this study, there were 8 one-dimensional demands, including professional psychology courses, professional psychology online counseling, professional psychology tests, stress relief games, light music, white noise, user privacy and security protection, and nurse confession. These demands not only constituted the key elements of digital mental health support, but also were all in the predominance area I in the importance-satisfaction matrix diagram. This distributional feature suggested that the nurse community’s expectations of these mental health supports were significantly higher than their actual experiences, and that there was a clear demand gap. While expecting professional support, nurses wanted to relieve their stress and emotions in a relaxing and enjoyable way, and had a strong desire for privacy and security in the process. This is expected to lead to a significant increase in nurse satisfaction if it is met in a timely manner. In digital mental health support scenarios, privacy security has become an important factor influencing participants’ willingness to use (35). Studies have shown that most nurses with psychological problems are often reluctant to disclose their psychological problems due to professional reasons or a sense of shame (36), which makes privacy and security protection an important consideration when they use digital mental health services. Therefore, targeted optimization of these modules may become a leverage point to enhance the efficacy of the digital mental health support system for nurses. From the perspective of the Job Demands-Resources model (37), these features function as important resources that help nurses cope with the high demands of their work environment. By providing professional guidance, interactive tools, and privacy-protected channels, they directly equip nurses with skills and resources to manage stress and occupational burnout, facilitate emotional regulation, and offer timely support when needed. Through the introduction of evidence-based design of professional courses, assessment tools that match clinical scenarios, and music therapy that incorporates neuroscience principles, the clinical adaptability of the service may be systematically enhanced, thereby better meeting the core demands of the nurses.

### 4.3 Attractive attributes

Attractive attributes are those that can surprise, referring to service items that have no expectations and do not cause dissatisfaction when they are not fulfilled, but additional fulfillment can be a surprise (38). In this study, there were 7 demands for attractive attributes, including intelligent mood monitoring, mindfulness practice, gratitude practice, muscle relaxation training, mental journaling, health monitoring, and live-streaming training. These 7 demands were located in the second quadrant of the matrix, and although the satisfaction coefficient was high, the importance was not strong enough and belonged to improving area II. It was clear from the survey results that the nurse community wanted a combination of physiological indicators, smarter monitoring and more effective interventions. Their desire was not directed at the complexity of the functionality itself, but rather focused on the ability of technology to transform their fragmented, high-pressure work situations into psychological support portals that could be called upon instantly. Analyzing the reasons, on the one hand, these items did not fall into the rigid list of daily or current psychological support in hospitals, and nurses did not take them for granted or should be equipped with them before completing the questionnaire; on the other hand, the attractive attributes were closely related to the specificity of nurses’ occupational context. The long-term high-pressure, fragmented work rhythm makes nurses extremely sensitive to the “cost of time” (39), and traditional psychological interventions are difficult to penetrate into the clinical front line because they take up a continuous period of time (40), while the demand for such attractive attributes can precisely match the work rhythm of nurses. At the same time, with the increasing development of live broadcasting, live broadcasting courses can increase the participation of nurses (41), and the fulfillment of this demand can further increase the sense of participation of nurses. This aligns with the broaden-and-build theory of positive emotions, which posits that positive emotions expand individuals’ thought-action repertoires and build enduring personal resources that contribute to wellbeing (42). According to this theory, mindfulness practices, gratitude exercises, and emotion regulation tools cultivate positive emotions, helping nurses not only cope with stress but also enhance their psychological resilience (43). Empirical studies have shown that gratitude interventions targeting nurses can significantly improve their psychological resilience and subjective wellbeing (44).

It is worth noting that attractive attributes and one-dimensional attributes are not static. According to the Kano type’s life cycle I → A → O → M (45), attractive attributes can be transformed into one-dimensional attributes to a certain extent. With the popularization of technology and the improvement of occupational mental health perception, the demand will change with time, and time will also affect the functional demand, and the service content of Kano type will be adjusted accordingly. Therefore, under the premise of meeting nurses’ must-be and desired demands, nurse managers can establish intelligent monitoring methods and effective interventions, pay continuous attention to the changes in nurses’ demands, and continuously enrich the way of nurses’ mental health support in order to adapt to the dynamic development of demands.

### 4.4 Indifferent attributes

Indifferent attributes are demands that have a small impact on importance and satisfaction and do not affect participants much whether they are provided or not. In this study, three demands were classified as indifferent attributes: psychological radio station, AI interaction technology, and check-in reward system. Regarding the psychological radio station, its placement may reflect a mismatch between content format and nurses’ actual usage scenarios. While audio-based interventions are effective in general populations (46), nurses in high-pressure environments may lack opportunities for sustained, focused listening.

The classification of AI interaction technology as an indifferent attribute warrants particular attention. Although AI is innovative in digital mental health systems, nurses did not demonstrate strong reliance on or expectation for this feature in their high-pressure work routines. Several factors may explain this finding. First, complex operation and cognitive burden in high-stress clinical environments may outweigh potential benefits (47). Second, low alignment with actual clinical scenarios means many AI tools fail to address nurses’ specific occupational stressors such as shift work and moral distress. Third, nurses’ limited artificial intelligence literacy may also contribute to this indifferent evaluation. Research indicates that nurses’ AI literacy is at a medium level, with relatively weak competencies in practical application (48, 49). This literacy gap could lead to underutilization of AI features, resulting in perceptions that they add little value. The check-in reward system, although it can incentivize nurses to maintain the habit of using it, may additionally increase the psychological burden of nurses if they are required to sign in frequently. These three demands were in the secondary improving area III, with small importance and satisfaction index values, and could be placed at the bottom of the list for improvement. Managers who plan to follow up with optimization should consider streamlining or adjusting these demand modules to avoid unnecessary functionality that places an additional burden on nurses and to allow more of their limited resources to be devoted to more critical areas of demand.

### 4.5 Comparison with prior work

Compared to traditional in-person mental health support for nurses, this study uses the Kano model to systematically categorize nurses’ needs into a hierarchical structure: must-be attributes (foundational requirements), one-dimensional attributes (performance-sensitive features), attractive attributes (potential delight factors), and indifferent attributes (low-priority elements). This stratified framework enables digital systems to allocate resources efficiently by prioritizing foundational features first, then progressively incorporating value-added functionalities based on their impact on user satisfaction. A review of existing literature indicates that previous studies on mental health interventions for nurses have primarily concentrated on validating the effectiveness of individual intervention methods or conducting general needs assessments, such as offline group counseling (50), mindfulness-based positive psychology interventions (51), and developing humanized management strategies. However, while these studies establish the need for interventions, they are often limited by broad, non-specific needs assessments and a lack of clear priority hierarchies. Future interventions should more precisely account for the distinct needs and unique pressures faced by nursing professionals (52).

These findings align with recent high quality international randomized controlled trials. For example, a Mexican study of 2,315 health care workers (53), grounded in the theory that mental health skills can be strengthened through psychoeducation and contemplative practice, showed that digital health training leads to significant reductions in distress and enhancements in wellbeing, with sustained effects over time. Similarly, a study conducted in Germany found that digital resilience training substantially enhanced psychological resilience and decreased perceived stress (54). These results align with the needs identified in the current study, including emotional regulation training and mindfulness practice, further validating the practical value of such digital interventions. The attractive attributes identified in this study, such as intelligent emotion monitoring and live-streaming training, reflect promising directions for the evolution of intervention models. These features align with global advancements in AI assisted mental health support (55), yet remain notably underexplored specifically among nursing populations. The hierarchical classification generated by this study provides a foundation for translating these findings into concrete design principles.

### 4.6 Technical implications

Based on the hierarchical classification generated by this study, we propose several technical design implications for digital mental health platforms targeting nurses, with a clear focus on development priorities. The foundational priority lies in must-be attributes, which function as basic support mechanisms. For digitally delivered psychological services, development efforts should concentrate on content quality, clinical accuracy, and relevance to nursing practice; for core platform features, the focus must be on usability, data security, and system reliability. These foundational elements are essential for establishing user trust and ensuring platform adoption. Building on this foundation, one-dimensional attributes should be developed as a core functional layer to enhance user satisfaction, serving as important job resources. Features such as professional counseling and stress relief tools can be designed to deliver content based on real-time stress levels, facilitating nurses’ psychological recovery, with investment in these features yielding proportional returns in user satisfaction. To further enhance system value, attractive attributes can be incorporated as a value-added layer to differentiate the platform and foster long-term engagement, functioning as broaden-and-build theory mechanisms. Mindfulness and emotion regulation tools may be offered as optional, user-driven modules, gradually helping nurses build psychological resilience and enhancing long-term user loyalty. It is equally important to recognize indifferent attributes, which represent features with limited return on investment. These features are not inherently problematic but fail to align with nurses’ actual work contexts and cognitive needs; developers should avoid adding unnecessary functionality that may increase burden rather than create value. Together, these three priority levels, foundational safety (must-be), core functionality (one-dimensional), and value-added innovation (attractive), provide a clear roadmap for developing digital mental health systems that comprehensively address nurses’ occupational mental health needs, while the identification of indifferent attributes serves as a crucial reminder to avoid resource misallocation.

## 5 Limitations

This study has several limitations. First, the cross-sectional design identified nurses’ needs but did not examine the influence of demographic factors, and the sample was restricted to tertiary hospitals in China, limiting generalizability to other settings. Second, the pilot study was primarily designed to assess questionnaire clarity and did not test the relevance, reliability, or construct validity of the proposed variables. Third, longitudinal studies are recommended to track how nurses’ needs evolve over time, providing a more robust evidence base for future iterations. Future research should address these limitations by expanding recruitment to diverse healthcare settings, employing longitudinal designs to track changes in nurses’ needs over time, and incorporating rigorous validation procedures to further establish the psychometric properties of the instrument.

## 6 Conclusion

Using the Kano model as the core analytical tool, this study systematically classifies nurses’ demands for digital mental health support, and clarifies the specific content and characteristics of must-be, one-dimensional, attractive, and indifferent demands. This refined classification based on demand attributes not only provides clear guidelines for identifying the key items that should be prioritized, but also avoids blind investment of resources. It can also accurately match nurses’ demands for psychological support in high-pressure work situations through in-depth analysis of different demand types, thus providing more targeted and efficient digital mental health support and scientifically constructing a precise psychological support system. Nursing administrators can prioritize and meet all types of demands to reduce nurses’ psychological pressure and enhance job satisfaction, laying the foundation for cultivating a high-level nursing workforce and promoting the high-quality development of nursing.

## Data Availability

The raw data supporting the conclusions of this article will be made available by the authors, without undue reservation.
