# Supplementary material for: Nurses’ needs for digital mental health support: an analysis based on the Kano model
Source: Front Public Health. 2026 Apr 21;14:1796306. doi: 10.3389/fpubh.2026.1796306 (PMC13139741; doi:10.3389/fpubh.2026.1796306)
Supplement: Supplementary file 1 [file Table_1.DOCX]

**Kano model questionnaire**

This questionnaire aims to assess your information needs for digital mental health. Each section consists of two questions.

1. **Positive question**: How do you feel when this service is provided?
2. **Negative question:** How do you feel when this service is not provided?

Please complete it based on your actual circumstances.

**Option Description:**

(1) “Like” means “I like it this way”.

(2) “Must be” means “it must be that way”.

(3) “Neutral” means “I am neutral”.

(4) “Live with” means “I can live with it that way”

(5) “Dislike” means “I dislike it that way.”

| No | Item | Question | Like | Must be | Neutral | Live with | Dislike |
| --- | --- | --- | --- | --- | --- | --- | --- |
| **1** | Intelligent emotion monitoring | provided |  |  |  |  |  |
|  |  | not provided |  |  |  |  |  |
| **2** | Professional psychology courses | provided |  |  |  |  |  |
|  |  | not provided |  |  |  |  |  |
| **3** | Professional psychology online counseling | provided |  |  |  |  |  |
|  |  | not provided |  |  |  |  |  |
| **4** | Professional psychology testing | provided |  |  |  |  |  |
|  |  | not provided |  |  |  |  |  |
| **5** | Psychological test result analysis | provided |  |  |  |  |  |
|  |  | not provided |  |  |  |  |  |
| **6** | Recommended psychological knowledge | provided |  |  |  |  |  |
|  |  | not provided |  |  |  |  |  |
| **7** | Psychology articles/educational videos | provided |  |  |  |  |  |
|  |  | not provided |  |  |  |  |  |
| **8** | Offline psychological counseling | provided |  |  |  |  |  |
|  |  | not provided |  |  |  |  |  |
| **9** | Mindfulness practice | provided |  |  |  |  |  |
|  |  | not provided |  |  |  |  |  |
| **10** | Emotional regulation training | provided |  |  |  |  |  |
|  |  | not provided |  |  |  |  |  |
| **11** | Stress relief games | provided |  |  |  |  |  |
|  |  | not provided |  |  |  |  |  |
| **12** | Gratitude practice | provided |  |  |  |  |  |
|  |  | not provided |  |  |  |  |  |
| **13** | Muscle relaxation training | provided |  |  |  |  |  |
|  |  | not provided |  |  |  |  |  |
| **14** | Mental journaling | provided |  |  |  |  |  |
|  |  | not provided |  |  |  |  |  |
| **15** | Light music | provided |  |  |  |  |  |
|  |  | not provided |  |  |  |  |  |
| **16** | White noise | provided |  |  |  |  |  |
|  |  | not provided |  |  |  |  |  |
| **17** | Psychological radio station | provided |  |  |  |  |  |
|  |  | not provided |  |  |  |  |  |
| **18** | Personal data recording | provided |  |  |  |  |  |
|  |  | not provided |  |  |  |  |  |
| **19** | User privacy and security protection | provided |  |  |  |  |  |
|  |  | not provided |  |  |  |  |  |
| **20** | Nurse peer support | provided |  |  |  |  |  |
|  |  | not provided |  |  |  |  |  |
| **21** | Voice sharing | provided |  |  |  |  |  |
|  |  | not provided |  |  |  |  |  |
| **22** | AI interaction | provided |  |  |  |  |  |
|  |  | not provided |  |  |  |  |  |
| **23** | Check-in reward | provided |  |  |  |  |  |
|  |  | not provided |  |  |  |  |  |
| **24** | Health monitoring | provided |  |  |  |  |  |
|  |  | not provided |  |  |  |  |  |
| **25** | Program customization | provided |  |  |  |  |  |
|  |  | not provided |  |  |  |  |  |
| **26** | Live-streaming training | provided |  |  |  |  |  |
|  |  | not provided |  |  |  |  |  |
